# Spatial distribution of breast cancer in Sudan 2010-2016

**DOI:** 10.1371/journal.pone.0211085

**Published:** 2019-09-16

**Authors:** Marwa Maweya Abdelbagi Elbasheer, Ayah Galal Abdelrahman Alkhidir, Siham Mohammed Awad Mohammed, Areej Abuelgasim Hassan Abbas, Aisha Osman Mohamed, Isra Mahgoub Bereir, Hiba Reyad Abdalazeez, Mounkaila Noma

**Affiliations:** 1 Department of Histopathology & Cytology, Faculty of Medical Laboratory Sciences, Alzaiem Alazhari University, Khartoum, Sudan; 2 Sudanese Medical Council, Khartoum, Sudan; 3 Zanam Specialist Hospital, Khartoum, Sudan; 4 Sudanese Medical Specialization Board, Khartoum, Sudan; 5 Sudanese National Council for Medical & Health Professions, Khartoum, Sudan; 6 University of Medical Sciences and Technology, Khartoum, Sudan; Kaiser Permanente Washington Health Research Institute, UNITED STATES

## Abstract

**Background:**

Breast cancer is the most prevalent cancer among females worldwide including Sudan. The aim of this study was to determine the spatial distribution of breast cancer in Sudan.

**Materials and methods:**

A facility based cross-sectional study was implemented in eighteen histopathology laboratories distributed in the three localities of Khartoum State on a sample of 4630 Breast Cancer cases diagnosed during the period 2010–2016. A master database was developed through Epi Info^TM^ 7.1.5.2 for computerizing the data collected: the facility name, type (public or private), and its geo-location (latitude and longitude). Personal data on patients were extracted from their respective medical records (name, age, marital status, ethnic group, state, locality, administrative unit, permanent address and phone number, histopathology diagnosis). The data was summarized through SPSS to generate frequency tables for estimating prevalence and the geographical information system (ArcGIS 10.3) was used to generate the epidemiological distribution maps. ArcGIS 10.3 spatial analysis features were used to develop risk maps based on the kriging method.

**Results:**

Breast cancer prevalence was 3.9 cases per 100,000 female populations. Of the 4423 cases of breast cancer, invasive breast carcinoma of no special type (NST) was the most frequent (79.5%, 3517/4423) histopathological diagnosis. The spatial analysis indicated as high risk areas for breast cancer in Sudan the States of Nile River, Northern, Red Sea, White Nile, Northern and Southern Kordofan.

**Conclusions:**

The attempt to develop a predictive map of breast cancer in Sudan revealed three levels of risk areas (risk, intermediate and high risk areas); regardless the risk level, appropriate preventive and curative health interventions with full support from decision makers are urgently needed.

## Introduction

Breast cancer (BC) is a disease characterized by different pathologies, biological characteristics and clinical behaviors. It is the leading cancer among females worldwide with 641,000 cases reported in 1980 and 1,643,000 cases in 2010; the annual incidence increase between the two years was 3.1% [1]. In the year 2015 the WHO reported 571000 deaths from BC [2] while in 2018 it represented 24.2% of all cancers and 15% of deaths due to cancer among females [3]. Furthermore by the year 2020, 1.7 million new cases are expected mostly in the developing countries [4]. The recent shift in its burden in the developing world is revealed by a high mortality rate and a poorer overall survival [2, 4]. The geographical distribution of BC in Africa revealed a marked variation in incidence within the continent with a high incidence rate of 130 cases per 100,000 in Northern African countries and a lowest rate of 95 cases per 100,000 recorded in the Western part of the African continent [5]. The highest standardized mortality rate worldwide according to WHO six regions was found in the East Mediterranean Office (EMRO) and Africa Regional Office (AFRO) with respectively 18.6% and 17.2% [6]. On the other hand breast cancer among males is still considered a rare condition, representing 1% of the total breast cancer patients in Europe compared to over 6% in Central African countries [7].

Sudan is third largest African country in terms of geographical range with an estimated population of 41 million, of which 33.7% were reported living in urban areas. Health services in Sudan are provided by the Federal and State Ministries of Heath, military, police, universities, and private sectors. There are over 100,000 health workers in Sudan with a doctor to nurse ratio of 1:2.5 as reported in 2013. Sudan has one physician for every 3,333 people according to a World Bank report, placing the country within the critical shortage zone according to the WHO criteria of 2.28 health care professionals per 1000 population. There is an inequitable and uneven geographical distribution of the health workforce in Sudan. According to the 2006 survey report, nearly 70% of health personnel work in urban settings serving about 30% of the total country’s population and more than one-third of the total health workforce was located in the capital city. Around 67% of health worker staff is working in secondary and tertiary facilities, as opposed to only 33% in primary healthcare settings [8]. There are three public cancer centers in Sudan; the Radiation Isotope Center Khartoum (RICK), Gezira Institute for Cancer treatment and Molecular Biology (GICMB), and Shendi Cancer Center [9].

In Sudan, the burden of cancer had increased from 303 cases in 1967 to 6303 in 2010 in which BC represented the most common cancer [10]. Further studies [11, 12] reported that the highest prevalence of cancers was recorded in the States of Khartoum, North Kordufan, Nile River, Northern, Gezira and White Nile states and BC was the most prevalent. According to the records of the Radiation Isotope Center Khartoum (RICK) and Gezira Institute for Cancer treatment and Molecular Biology (GICMB), BC was the most predominant malignancy among females with respectively 29–34.5% and 30.0% of the cancers registered. Most cases were young-aged women. About 40% were below 45 years (mean age of 50) with late advanced disease. On the other hand, male cancer constituted 3.5–4% [10, 13]. Furthermore, studies from Red Sea State (2003–2006) and Central Sudan (1999–2006) revealed that the majority of the patients were premenopausal women (age <50 years) who presented with a late stage metastasized disease [14, 15].

A study [16] was conducted based on 6771 cases of cancers diagnosed in Khartoum State by Sudan First National Cancer registry during the period 2009 to 2010. The findings revealed that the most common cancer was breast cancer with an incidence rate of 25.1 per 100,000. The study also reported the possibility of underestimation of the affliction which could be due to factors such as stigmatization and poverty, leading to undiagnosed or untreated cases. Overestimation was also pointed out for elderly patients who might be treated symptomatically at primary care levels or died before reaching cancer specialized institutions.

Currently there are no significant ongoing activities in cancer control at the primary level, and screening, diagnostic, and therapeutic services are only being provided as secondary and tertiary care, indicating a weak referral system and links [9]. In addition to that there is a shortage in the number of cancer centers in Sudan, fallings below the recommendations set by the International Atomic Energy Agency (IAEA) [17], which recommends one center for every 2–5 millions of population. Furthermore the public cancer centers are clustered in the central states of Sudan which limits access for patients living in the peripheral parts of the country [9].

Available statistics on breast cancer in Sudan are mostly restricted to central institutions such as RICK and GICMB and the geographical distribution of the disease yet is unknown. This paper aimed to estimate the prevalence of breast cancer and determine its spatial distribution country-wide.

## Methods

### Ethical statement

This study’s ethical clearance was obtained from Khartoum State Ministry of Health, Directorate of innovation and Scientific research Ethical Committee on 11th May 2017 following the approval of the research proposal discussed with the Research Review Board of Scientific Research of The Ministry. The committee waived the need of consent from the patients based on the following: Some laboratories registration system lacks information related to contact phone numbers hence then the consent will not be applicable for those patients.

Personal information of the patients were collected to enable access to the patients for the community phase and to avoid duplicate of patients across the histopathology laboratories as patients could use more than one histopathology laboratory. Both the Ministry and the histopathology laboratories were fully aware of the importance to have a Breast Cancer Registry and all the data extracted have a unique identification code.

Verbal consent was obtained from each of the patients involved in the community survey (manuscript being drafted to submit for publication). The verbal consent was obtained from each participant through a telephone interview after introducing the topic and explaining to the participants their full freedom to participate or not in the study and their right to not answer any of the questions they didn’t wish to do so. Hence we have obtained the verbal consent of all the patients who were included in the community survey. For those who were lost, a co-patient freely accepted to take the interview following a well informed consent".

### Study design and population

**Fig 1:**
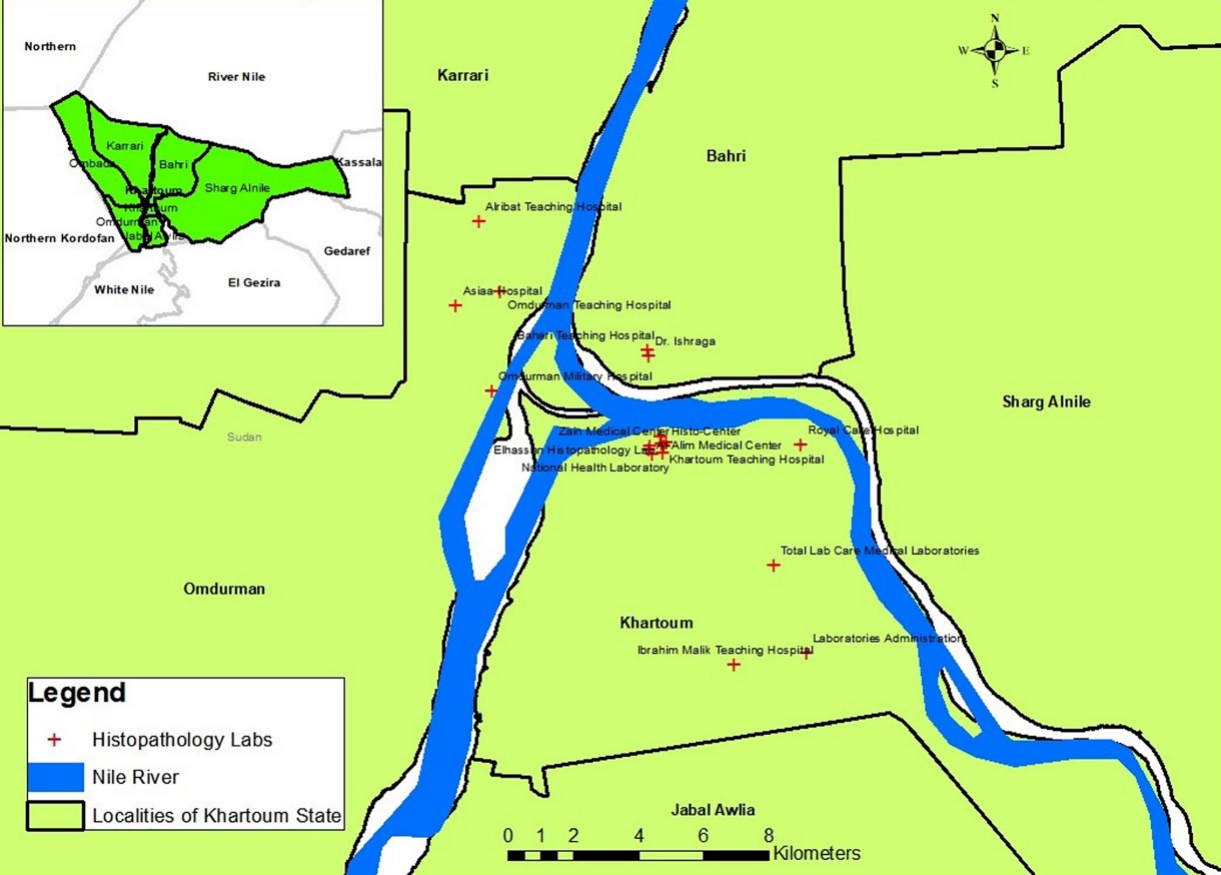
Geographical distribution of the histopathology laboratories. The geographical information system ArcGIS 10.3 for Desktop version 10.3.0.4322 was used to generate “Fig 1”. Sudan administrative divisions were downloaded from http://www.maplibrary.org/library/stacks/Africa/Sudan/index.htm, and uploaded in ARCGIS. All the administrative divisions of Sudan were first displayed as a layer. Then the limits of the layers corresponding to Sudan States were selected and saved. When the map was elaborated, the features of ArcGIS were used to convert the map into the figure. The capacity in geographical information system was introduced from 2015 in the University of Medical Sciences and Technology, Khartoum, Sudan by Dr. Mounkaila Noma [18].

A facility based cross-sectional study was implemented. Data were extracted from eighteen histopathology laboratories within Khartoum State (Fig 1). In each of these laboratories, data collected included facility name, type (public or private), and geo-location (latitude and longitude). Personal data extracted from the facility records were name, age, marital status, ethnic group, state, locality, administrative unit, permanent address and phone number. Other information obtained from the records included the date of diagnosis and the histopathology diagnosis. Information regarding ethnic and socioeconomic status of the patients was excluded during analysis due to the considerable amount of missing data from the records.

### Data management and analysis

The master database, consisting of 4630 patient medical records was developed through Epi Info^TM^ 7.1.5.2 and thereafter filtered through the statistical package for social sciences (SPSS version 23) to exclude cases lacking important information such as the histopathology diagnosis, and date of diagnosis, as well as duplicated cases which were entered twice. The data of the remaining 4423 records was then summarized through SPSS to generate the frequency distribution of the cases in terms of person (age, gender) and type of cancer diagnosed by the histopathology centers. Histopathology diagnoses recorded were invasive ductal carcinoma, invasive lobular carcinoma, carcinoma in situ and others, which were then regrouped to fit WHO 2012 classification [19]. The epidemiological distribution of breast cancer in Sudan was based on 1135 records for which data on residence were available. Those 1135 records were geo-referenced to facilitate the plotting of the residence of the patients. Prevalence was estimated using the updated 2016 Sudan Census Bureau and Statistics population data as a reference. ArcGIS 10.3 spatial analysis features were used to develop a risk map based on the kriging method [20].

The geographical information system, ArcGIS 10.3 for Desktop version 10.3.043322, was used to generate the geographical distribution and risk maps of breast cancer. The risk map of Cancer was developed by using the *kriging method of data interpolation*. This method is based on the *semivariogram* which captures the spatial dependence between samples by plotting semivariance against separation distance. The premise of any spatial interpolation is that close samples tend to be more similar than distant samples (*this is also called spatial autocorrelation*). This property of spatial data is implicitly used in inverse distance weighted (IDW) interpolation to determine cell values using a linearly weighted combination of a set of sample points. Inverse distance weighted (IDW) is a method of interpolation that estimates cell values by averaging the values of sample data points in the neighborhood of each processing cell. The closer a point is to the center of the cell being estimated, the more influence, or weight; it has in the averaging process. The weight is a function of inverse distance. The surface being interpolated should be that of a location dependent variable. In kriging, one must model the spatial autocorrelation using a semivariogram instead of assuming a direct, linear relationship with separation distance [20].

## Results

**Fig 2:**
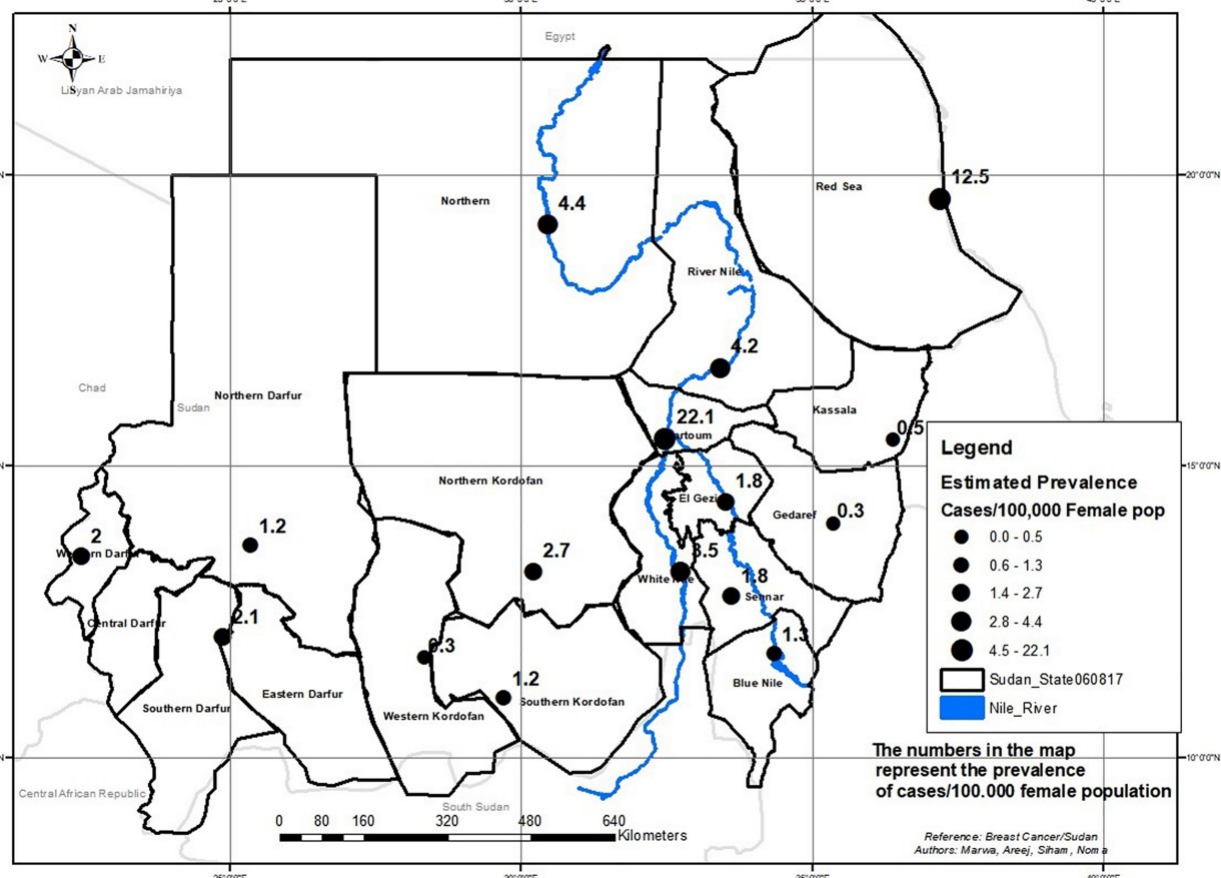
Epidemiological distribution of Breast Cancer in female population in Sudan (n = 1135). The geographical information system ArcGIS 10.3 for Desktop version 10.3.0.4322 was used to generate “Fig 2”. Sudan administrative divisions were downloaded from http://www.maplibrary.org/library/stacks/Africa/Sudan/index.htm, and uploaded in ARCGIS. All the administrative divisions of Sudan were first displayed as a layer. Then the limits of the layers corresponding to Sudan States were selected and saved. When the map was developed, the features of ArcGIS were used to convert the map into the figure. The capacity of the geographical information system was introduced from 2015 in the University of Medical Sciences and Technology, Khartoum, Sudan by Dr. Mounkaila Noma [18].

A total of 4423 cases of breast cancer were recorded (2010–2016) from eighteen laboratories distributed in Khartoum State. Patients were aged 12 to 103 years with an average (median) age of 48 years. They were predominately females 97.4% (4300/4413). The mean age at presentation was higher in males (61 years ±14.9) than in females (49 years ±14.2). Of the 4423 cases of breast cancer, invasive breast carcinoma of no special type (NST) was the most frequent histopathological diagnosis (79.5%, 3517/4423) followed by special subtypes of invasive carcinoma (12.4%, 547/4423) and precursor lesions (3.2%, 142/4423) and the remaining 4.9% were classified as others. Females were paying the highest burden with a crude prevalence of 3.9 cases per 100,000 female population, ranging from 0.3 (Gedaref and Western Kordofan) to 22.1 in Khartoum as shown by Table 1 and Fig 2. On the other hand, male breast cancer was <1 per 100,000 male population.

**Table 1:** Crude Prevalence (cases/100,000 population) of Breast Cancer in Sudan, data from eighteen histopathology laboratories located in Khartoum State (n = 1135).

	Cases recoded from Labs			Prevalence per 100,000 population		Total population		SCBS
States	Number	Female	Male	Female*	Male**	Female	Male	2016
Khartoum	786	766	20	22.1	0.5	3,464,536	3,920,622	7,385,158
Red Sea	79	77	2	12.5	0.2	616,423	828,930	1,445,353
El Gezira	45	44	1	1.8	0.1	2,464,166	2,295,598	4,759,764
Northern Kordofan	45	44	1	2.7	0.1	1,628,070	1,512,107	3,140,177
White Nile	43	42	1	3.5	0.1	1,183,915	1,140,529	2,324,444
River Nile	30	29	1	4.2	0.1	699,980	729,533	1,429,513
Southern Darfur	27	26	1	2.1	0.1	1,244,275	1,299,942	2,544,217
Northern	20	19	1	4.4	0.1	438,160	448,851	887,011
Sennar	17	17	0	1.8	0.1	912,230	865,752	1,777,982
Northern Darfur	14	14	0	1.2	0.0	1,115,490	1,165,395	2,280,885
Blue Nile	7	7	0	1.3	0.0	517,492	531,874	1,049,366
Southern Kordofan	6	6	0	1.2	0.0	501,841	490,106	991,948
Western Darfur	6	6	0	2.0	0.1	285,234	273,108	558,342
Kassala	5	5	0	0.5	0.0	1,053,571	1,306,512	2,360,083
Gedaref	3	3	0	0.3	0.0	1,012,329	999,285	2,011,614
Western Kordofan	1	1	0	0.3	0.0	282,473	275,868	558,342
Rumbeck***	1							
**Total**	**1135**	**1105**	**29**	**3.9**	**0.1**	**17,420,186**	**18,084,011**	**35,504,197**

SCBS: Sudan Census Bureau and Statistics

* Crude prevalence computed as number of breast cancer cases/ 100,000 female total population

** Crude prevalence computed as number of breast cancer cases/ 100,000 male total population

*** In Lakes State of South Sudan

**Fig 3:**
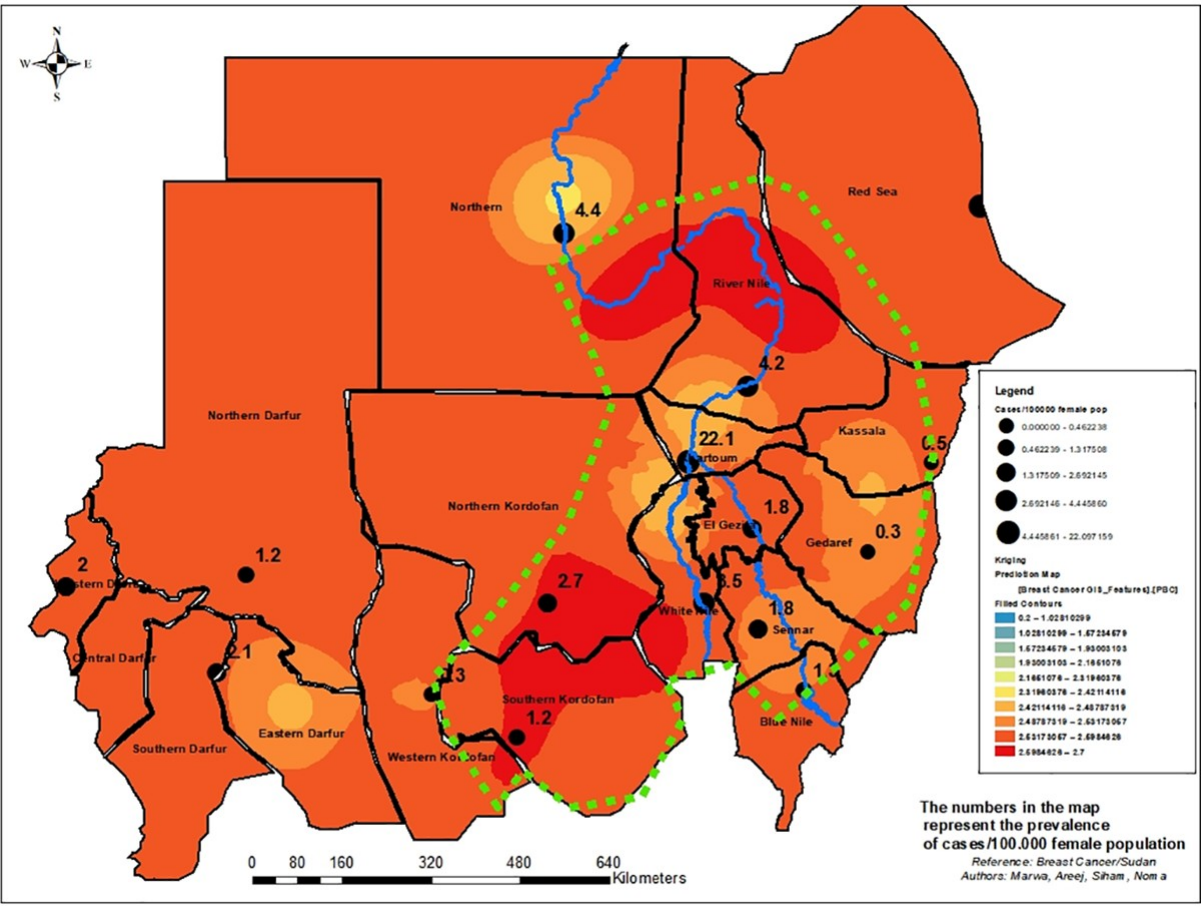
Breast Cancer risk map in Sudan (n = 1135). The geographical information system ArcGIS 10.3 for Desktop version 10.3.0.4322 was used to generate “Fig 3”. Sudan administrative divisions were downloaded from http://www.maplibrary.org/library/stacks/Africa/Sudan/index.htm, and uploaded in ARCGIS. All the administrative divisions of Sudan were first displayed as a layer. Then the limits of the layers corresponding to Sudan States were selected and saved. When the map was elaborated, the features of ArcGIS were used to convert the map into the figure. The capacity of the geographical information system was introduced from 2015 in the University of Medical Sciences and Technology, Khartoum, Sudan by Dr. Mounkaila Noma [18].

The spatial analysis confirmed that Breast Cancer is a country-wide health problem. The risk of breast cancer according to the map generated using the kriging method of the spatial distribution indicated three gradient scale colors of risk (Fig 3). Risk areas included Western, Central, Southern Darfur and partially Northern states, and a large part of Red Sea; Invasive carcinoma was the predominant type in those States. Intermediate risk areas, a mosaic for invasive carcinoma NST, Special Subtypes of Invasive Carcinoma and Precursor lesions, included the States of Khartoum, Gezira, White Nile, Kassala, Gedaref, Sennar, Eastern Darfur, and focally Northern Darfur. High risk areas were the States of Nile River, Northern, Red Sea (focal), White Nile, Northern and Southern Kordofan.

## Discussion

Our findings revealed a crude prevalence of 3.9 cases per 100,000 female populations for the period 2010 to 2016. This burden in Sudan females was also reported elsewhere in Sub-Saharan African countries, fluctuating from 4.5% (Zimbabwe) to 38.9/100 000 females in (South Africa) [21]. The crude prevalence of male breast cancer was 0.1 cases per 100,000 population which is much lower than figures reported by the Surveillance, Epidemiology and End Result (SEER) Program which showed that the rate of male breast cancer in the USA was 1.44 for 100,000 men in 2010 [7]

The average age of our female patients was 49 years revealing that younger population was affected as reported in Central Africa (45.83 years) and Ghana (49.1 years) [22, 23]. On the contrary in developed countries, women are affected at older age respectively at 57 years and 62 years in New Zealand and United States [24, 25]. The possible cause for this early predisposition of breast cancer among Sudanese females is still unknown. However some studies suggested some genetic factors which may contribute to this early onset of the disease [10, 26, 27].

Invasive carcinoma of NST was the prevalent type (79.5%) of breast cancer in our study as previously published in Sudan [13], elsewhere it was 60% of breast cancer cases as reported by Badowska-Kozakiewicz, et al. [28].

The Epidemiological map generated per states indicated that the highest prevalence was recorded in Khartoum and Red Sea States with respectively 22.1 and 12.5 per 100,000 female populations. The figures from Khartoum and Red Sea States may be interpreted as related to the fact that most of the cases were reported from those States. However we endorse the contrary based on the results of the modeling method for predicting the risk score, in which the prevalence from these two states in the inverse direct interpolation was weighted with the distance to provide a risk score which balance the prevalence in these two states. As such the spatial distribution predicted Khartoum State as intermediate risk whereas Red Sea State was displayed as a highly focal risk area. In the overall, we would like to emphasis that the breast cancer is a country wide public health problem. The delineated belt is a subject for further discussion related to the modeling technique and the limitations of the data which does not include environmental and socio-economic factors.

This rapid evidence-based delineation of breast cancer areas is a tool for guiding public health professionals and decision makers to establish a breast cancer program for fine tuning the epidemiological map and the subsequent risk map generated as applied in health sector in Iran [29] and elsewhere in Saudi Arabia [30] where the geographical information was used to set priorities.

A limitation of data collection was that records and histopathology reports were individually designed by the labs, and as such many labs overlook the inclusion of important socio-demographic and economic data in their records. This can be attributed to a recognizable weakness in the national Health Information Systems (HIS) which also suffers from the fact that large amounts of the community level information are not pooled into the HIS and that some programs collect and use data for their own activities and stop without disseminating their findings; in addition to the limited capacity for analysis, utilization, and dissemination of data and findings within the health system [8]

Our model was limited by the lack of environmental data to better assess the pattern of breast cancer which is a multi-factorial condition. Unfortunately, due to financial constraints the team was not able to collect the relevant environmental data (data from soil, surface and ground water, air) from high risk areas identified by the study to enable further strengthening of the model. The risk map was developed based on individual location of residence reported by the patients which may be a limiting factor leading to the over estimation or under estimation of the number of cases per State. This potential bias was minimized by the kriging method applied.

## Conclusion

Our findings provided an understanding of the pattern of the spatial distribution of breast cancer country wide with hot spots defined as high risk and intermediate risk areas. As further data may be needed to improve the risk map, the decision makers and the health professionals should for equity reasons look at decentralizing of the health system which could not be efficient and operational if all the expertise are concentrated mainly in the State hosting the capital of country.

## Supporting information

S1 File Breast Cancer dataset. (XLS)
